# Tissue-specific pathway activities: A retrospective analysis in COVID-19 patients

**DOI:** 10.3389/fimmu.2022.963357

**Published:** 2022-09-15

**Authors:** Nhung Pham, Finterly Hu, Chris T. Evelo, Martina Kutmon

**Affiliations:** ^1^ Department of Bioinformatics (BiGCaT), School of Nutrition and Translational Research in Metabolism (NUTRIM), Maastricht University, Maastricht, Netherlands; ^2^ Maastricht Centre for Systems Biology (MaCSBio), Maastricht University, Maastricht, Netherlands

**Keywords:** SARS-CoV-2, tissue-specific proteomics data, pathway activity, WikiPathways, COVID-19

## Abstract

The ACE2 receptors essential for SARS-CoV-2 infections are expressed not only in the lung but also in many other tissues in the human body. To better understand the disease mechanisms and progression, it is essential to understand how the virus affects and alters molecular pathways in the different affected tissues. In this study, we mapped the proteomics data obtained from Nie X. *et al.* (2021) to the pathway models of the COVID-19 Disease Map project and WikiPathways. The differences in pathway activities between COVID-19 and non-COVID-19 patients were calculated using the Wilcoxon test. As a result, 46% (5,235) of the detected proteins were found to be present in at least one pathway. Only a few pathways were altered in multiple tissues. As an example, the Kinin-Kallikrein pathway, an important inflammation regulatory pathway, was found to be less active in the lung, spleen, testis, and thyroid. We can confirm previously reported changes in COVID-19 patients such as the change in cholesterol, linolenic acid, and arachidonic acid metabolism, complement, and coagulation pathways in most tissues. Of all the tissues, we found the thyroid to be the organ with the most changed pathways. In this tissue, lipid pathways, energy pathways, and many COVID-19 specific pathways such as RAS and bradykinin pathways, thrombosis, and anticoagulation have altered activities in COVID-19 patients. Concluding, our results highlight the systemic nature of COVID-19 and the effect on other tissues besides the lung.

## 1 Introduction

When SARS-CoV-2 is inhaled into the body, it binds to the Angiotensin-converting enzyme 2 (ACE2) receptors to enter cells where it alters and hijacks many cellular processes (1). The primary infection route is the lower respiratory tract. Patients with severe symptoms often experience significant damage to their lungs, which can lead to pneumonia and/or Acute Respiratory Distress Syndrome (ARDS) (1), which are the leading cause of death in COVID-19 patients (2).In fact, a reported death is only associated with COVID-19 if a positive tested patient developed pneumonia and/or ARDS (2, 3).

It should be emphasized that COVID-19 is not just a lung disease, but also a multisystem illness involving several organs (4–7). Injury to these organs can be caused directly by viral infection or secondary by organ dysfunction. Although the highest levels of SARS-CoV-2 copies per cell are detected in the respiratory tract (1, 8), the viral receptors, ACE2 proteins, are found with high levels in the small intestine, testis, kidney, heart, colon, and thyroid gland. In the lung and liver, ACE2 is moderately expressed. ACE2 is also found less abundant in the spleen and brain (9). SARS-CoV-2 has been detected with low copies per cell in the kidneys, liver, heart, brain, blood, and reproductive systems (1, 8)

Many COVID-19 patients died from multi-organ dysfunction in which respiratory, cardiac, and renal impairments were often observed (4, 9, 10). In the heart, the virus can cause acute coronary syndrome, congestive heart failure, myocarditis, and arrhythmias. Cardiac infection by SARS- CoV-2 was frequently found in autopsy cases (11). The possible mechanisms leading to the heart injury are direct myocardial injury by the virus through ACE2 entry, hypoxia-induced myocardial injury, microvascular damage and endothelial shedding, and cytokine/inflammation-mediated damage (12).

Kidney dysfunction is another common condition observed in severe, hospitalized COVID-19 patients (10, 13, 14). Kidney dysfunction is characterized by elevated levels of blood urea nitrogen, creatinine, uric acid, and D-dimer, along with proteinuria and hematuria (13). Severe acute kidney injury requiring renal replacement therapy. Kidney damage is usually the result of systemic abnormalities (15). Another common feature of severe COVID-19 is liver injury which is characterized by the increase of aspartate aminotransferase and alanine aminotransferase (16). Liver injury may be directly from viral infection or because of inflammation and metabolic abnormalities (16, 17). Metabolizing drugs by the liver and renal excretion of drugs is important for an efficient treatment. Damage in the liver and kidney can increase the risk of toxicity and influence the effect of medications (4, 17).

Effects of COVID-19 in other organs are less prominent, however, they are found to be associated with disease severity and may impose long-term effects. Thyroid and its hormones have been shown to affect the effectiveness of innate and adaptive immune systems. A hyperthyroid state is associated with more active responses of both the innate and adaptive immune systems (18). In COVID-19 patients, the level of triiodothyronine (TT3) was significantly reduced (19, 20). TT3 from the thyroid has a pro-inflammatory effect in immune cells such as neutrophils and macrophages. This decrease in TT3 level was positively correlated with the severity of COVID-19 (20). However, it is not yet clear if the changes in thyroid hormones may be important in COVID-19 severity and whether triiodothyronine supplementation could be a potential therapy (20, 21).

Current studies confirm that SARS-CoV-2 can also affect the male reproductive system. Testis impairment with spermatogenic dysfunction was observed in patients during and after recovery from COVID-19 (19). Severe inflammation damage was detected in testes and the presence of virus in testicular tissue was found in autopsy samples (22). These damages can be directly from viral infection or indirectly caused by COVID-19 immune responses (22–25).

The spleen and lymph nodes are other organs that are also affected by SARS-CoV-2 infection. Massive necrosis of the spleen and lymph nodes has been observed in some COVID-19 cases (26). These damages can be directly caused by viral infection (26–29).

However, the detailed molecular mechanism explaining the effects of SARS-CoV-2 in different tissues is still lacking. At molecular levels, proteomics can depict protein activity that can influence the activity of a pathway. This is due to the role of proteins in a pathway by interacting with or regulating other proteins, or as enzymes catalyzing reactions in metabolic pathways. Pathway models, machine-readable descriptions of a biological process that involves a cascade of interactions among molecules to produce a product or change in a cell can be useful to project the proteomics data. In this research, we aim to investigate the underlying molecular mechanisms of COVID-19 in different tissues using proteomics data and pathway models. We developed an automatic workflow to analyze and visualize multi-organ proteomics data to study the molecular pathway activity in COVID-19 patients.

## 2 Materials and methods

### 2.1 Dataset and pre-processing

A recently published multi-organ proteomics dataset by Nie X. et al. (2021) was used for the analysis (30). The data was obtained from autopsy samples from 19 COVID-19 and 56 non-COVID19 (cancer) patients in seven tissues: lung, spleen, liver, heart, kidney, testis, and thyroid. The annotated and normalized data provided in the Supplementary Material by Nie X et al. (2021) was used (30).

### 2.2 Pathway collection

In this study, the highly curated collection of pathway models from the COVID-19 Disease Map project (version 2021-06) was used (31) containing 21 pathways from MINERVA, WikiPathways (32), and Reactome (33). The collection was supplemented with 706 other non-COVID19 specific cellular pathway models from WikiPathways (version 2022-01-10), an established community-curated pathway database.

### 2.3 Pathway activity statistics and visualization

First, pathway activities in COVID-19 patients were calculated as the median protein abundance of all proteins in the pathway for each tissue separately.

Second, to study differences in pathway activities between COVID-19 and non-COVID-19 patients, a standard R package (stats) was used to perform a Wilcoxon rank-sum test comparing the protein expression distributions for each pathway in COVID-19 and non-COVID-19 patients. The effect size was calculated by dividing the absolute (positive) standardized test statistic z by the square root of the number of pairs. Only pathways with more than three proteins detected and at least 30% of the proteins measured in the pathway were included in the study. Pathways were considered altered in COVID-19 patients with a p-value < 0.05 and an effect size > 0.3. Next, selected pathways were visualized and analyzed using the open-source network analysis and visualization toolbox Cytoscape (34) with the WikiPathways app (35). The analysis is automated and implemented in R to increase reproducibility and reusability. The code with detailed documentation is available on GitHub (36).

## 3 Results

### 3.1 Protein-pathway statistics

**Figure 1:**
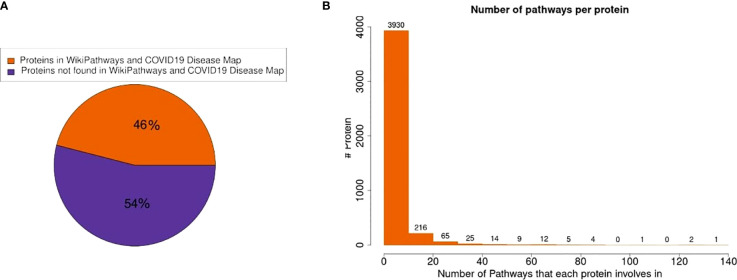
Protein presence in pathway models. **(A)** 46% of the measured proteins in the dataset were found in at least one pathway in the combined pathway collection. **(B)** The distribution of the number of pathways in which a protein participates shows that most proteins are only present in very few pathways.

In this study, we analyzed pathway activities using a published proteomics dataset from Nie X. et al. (2021) (30). The dataset measured proteins in COVID-19 and non-COVID-19 patients in seven tissues: the thyroid, lung, heart, kidney, liver, spleen and testis. The measured proteins in the dataset were mapped to the pathway collection from the COVID-19 Disease Map (31) and WikiPathways (32). Of the 11,384 detected proteins in the dataset, 5,235 (46%) proteins were present in at least one of the 727 pathways (**Figure 1**). Most proteins participate in only a few pathways (88% in less than 5 pathways, **Figure 1B**). Hub proteins participating in more than 100 pathways include major regulators like MAPK1 (135 pathways), AKT1 (130 pathways), NFKB1 (125 pathways), and MAPK3 (110 pathways). Nearly all pathways in the combined collection contained at least one of the measured proteins (723 out of 727 pathways). Of these, 640 pathways (88%) fulfill the criteria of more than three proteins and at least 30% measured proteins. These pathways were included in further analysis.

### 3.2 Pathway activities in COVID-19 patients

To assess pathway activities, we calculated the median expression of all protein abundances in the pathway for each tissue. We found many pathways from the COVID-19 Disease Map and the COVID-19 portal in WikiPathways such as the MINERVA Virus replication cycle, SARS-CoV-2 and COVID-19 pathway (WP4846) and nsp1 from SARS-CoV-2 inhibits translation initiation in the host cell (WP5027) among the most active pathways in COVID-19 patients. Besides COVID-19 specific pathways, we also identified high activity of many of the immune related pathways such as Type I interferon induction and signaling during SARS-CoV-2 infection (WP4868), TGF-beta signaling pathway (WP366), and IL-6 signaling pathway (WP364) in most tissues clearly indicating an ongoing immune response. In our control group of non-COVID-19 cancer patients, these pathways are also detected to be active. A full list of pathway activities is provided in the **Supplementary File 8**.

### 3.3 Pathways with changes in activity among tissues

**Figure 2:**
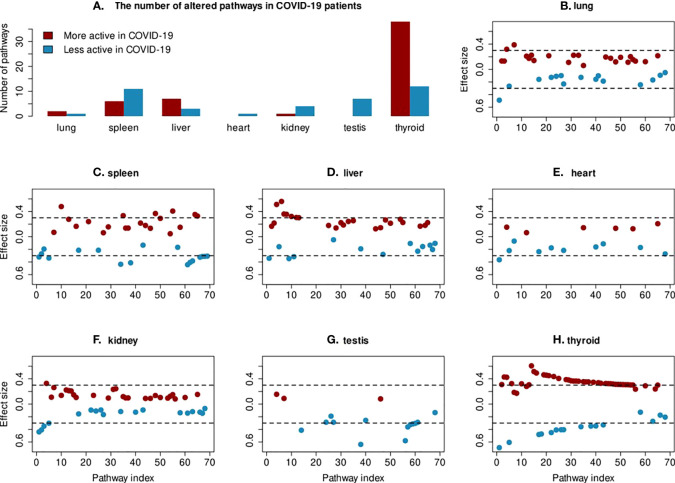
Changes in pathway activities in COVID-19 patients. **(A)** The box plot shows the number of less and more active pathways in each tissue. **(B–H)** For each tissue, the effect size of all significant pathways from the Wilcoxon test are shown. The cutoff of >0.3 for effect size is indicated with a dashed line. Only the 69 pathways, which show significant changes in protein abundance in at least one tissue are included on the x-axis.

**Figure 3:**
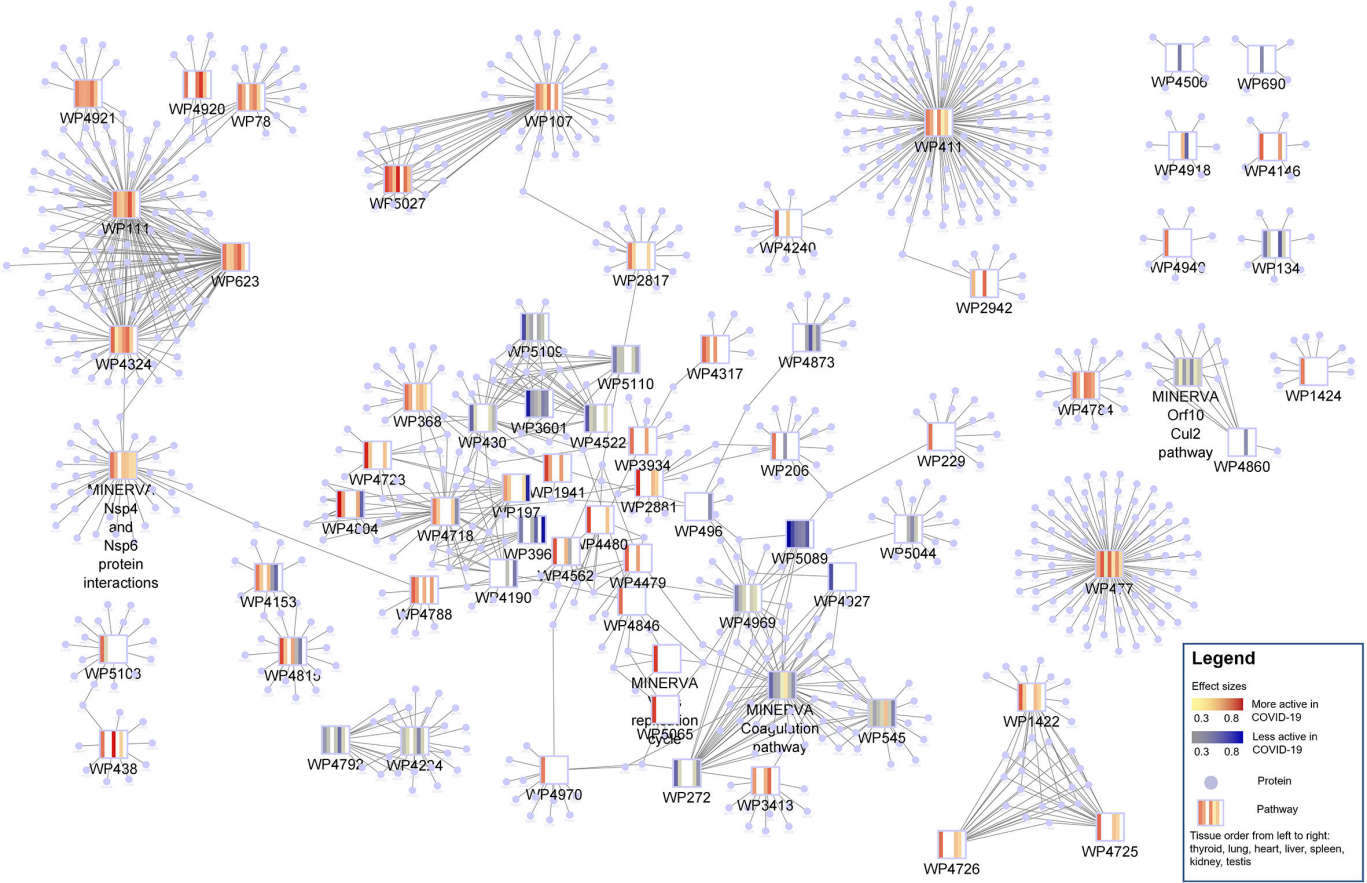
Protein-pathway network of 69 changed pathways in COVID-19 patients. Pathways are shown as rectangles and the proteins present in a pathway are connected to the pathway node and shaped as circles. The pathway nodes visualize the heatmap of pathway activity change in COVID-19 patients from thyroid, lung, heart, liver, spleen, kidney and testis in that order. Blue indicates a lower pathway activity in COVID-19 patients (protein abundance distribution shifted to the left) and red indicates an increased activity (protein abundance distribution shifted to the right). Most of the pathways are highly connected with many proteins in common.

Next, we focused on the differences in pathway activity between COVID-19 and non-COVID-19 patients to study specific changes in the tissues. The changes in pathway activities in COVID-19 patients were calculated using a Wilcoxon test comparing protein abundance distribution between COVID-19 patients and non-COVID-19 patients in each pathway. Pathways with a significant difference and effect size > 0.3 were selected for further visualization and analysis. In total, 69 out of 640 pathways were found to have altered activities in at least one tissue (**Figure 2**). A full list of changed pathways is provided in the **Supplementary Tables 1–7**. Among tissues, the thyroid has the greatest number of pathways that change activities in COVID-19 patients (**Figure 2**). Most of the changed pathways are highly connected with each other with many proteins in common (**Figure 3**). Most pathways that have their activities changed in COVID-19 deceased patients are metabolism of sphingolipid and cholesterol, virus entry and replication, inflammation regulatory pathways, electron transport chain, and mevalonate pathways. Most of the immune response pathways often reported for COVID-19 do not show changed activity including type 1 interferon signalling pathway (WP4868), TGF-beta signalling pathway (WP366), IL-1 signalling pathway (WP2332), IL-6 signalling pathway (WP364), Toll-like receptor signaling related to MyD88 (WP3858), TNF-related weak inducer of apoptosis (TWEAK) signaling pathway (WP2036), or p38 MAPK signaling pathway (WP400). Although these pathways contain differentially expressed proteins, the majority of proteins in these pathways do not change which make the overall activities of these pathways remain stable.

### 3.4 Pathway activities overlap between tissues

**Figure 4:**
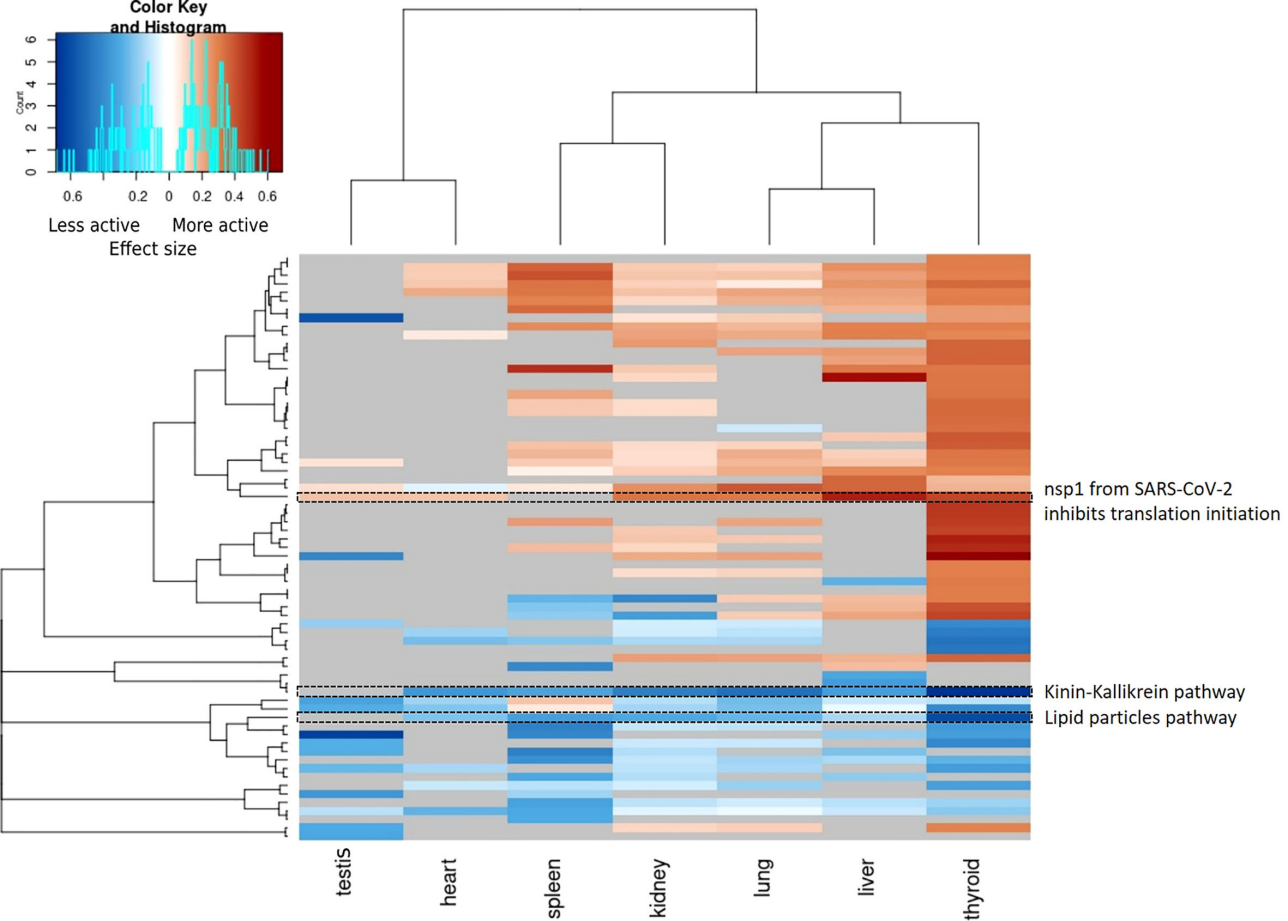
Heatmap of pathway activities per tissue (p-value < 0.05, Wilcoxon test). Pathway activities are highly tissue specific and only few pathways show significant changes in multiple tissue. All significant changed pathway are shown and the color gradient indicates the effect size. Blue: the pathway is less active in COVID-19 patients, red: the pathway is more active in COVID-19 patients. Grey: unmeasured or non-significant pathways (p-value >= 0.05).

**Figure 5:**
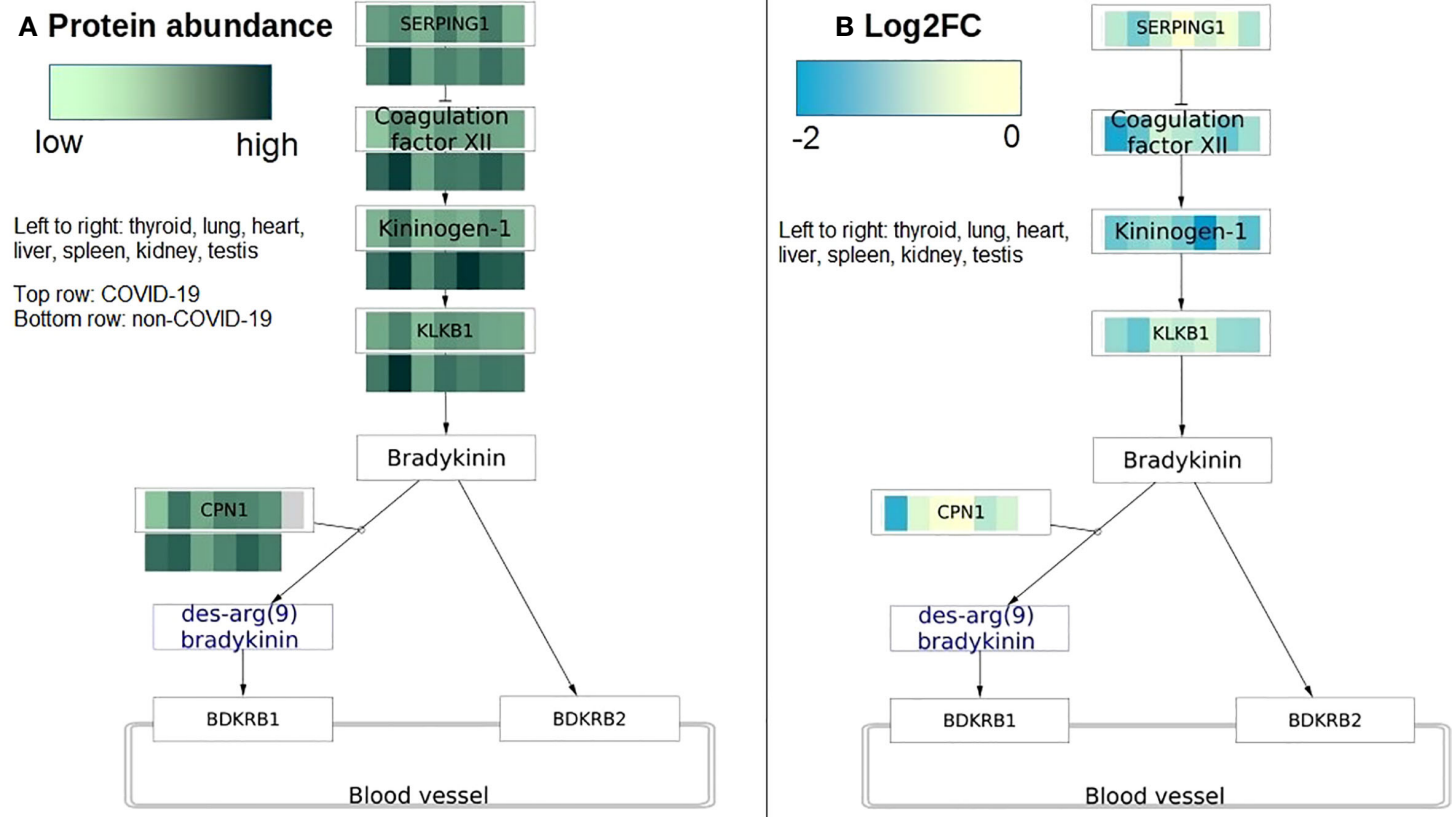
COVID-19 protein expression visualized on the Kinin Kallikrein pathway (WP5089). The pathway has activities changed in most tissues in COVID-19 patients. **(A)** Protein abundance is visualized on protein nodes in the pathway. Top row is data from COVID-19 patients and bottom row is data from non-COVID-19 patients. The higher the abundance the darker the value. The data nodes are split in seven columns for the seven different tissues. **(B)** The pathway figure shows the differential expression of the proteins (log2 fold change) between COVID-19 and non-COVID-19 patients. All proteins are downregulated or not changed in all tissues. The data nodes are split in seven columns for the seven different tissues. Left to right: thyroid, lung, heart, liver, spleen, kidney, testis.

In general, changes in pathway activity between COVID-19 and non-COVID-19 are highly tissue specific (**Figure 4**). While we do see some regular patterns of increased and decreased activity across tissues, only few pathways are altered in multiple tissues, e.g., the Kinin-Kallikrein pathway (WP5089) showed less activity in six organs: the thyroid, lung, spleen, liver, kidney, and heart (**Figure 5**). All proteins in the Kinin-Kallikrein pathway are less abundant in these tissues in COVID-19 patients. The pathway “nsp1 from SARS-CoV-2 inhibits translation initiation in the host cell” (WP5027) is more active in four tissues (thyroid, lung, liver, and kidney). The lipid particles composition (WP3601) pathway was less active in three tissues: the thyroid, spleen, and kidney. There are 15 pathways that change in more than one tissue, see **Table 1**. Most of them are related to energy pathways such as Electron transport chain: OXPHOS system in mitochondria, Oxidative phosphorylation, Mitochondrial complex III assembly, Mevalonate pathway and Pentose phosphate metabolism; lipid pathways such as cholesterol synthesis, Lipid particles composition, and degradation pathway of sphingolipids.

**Table 1:** Pathways with changed protein activity in COVID-19 patients in more than one tissue.

Pathway	Thyroid	Lung	Heart	Kidney	Liver	Spleen	Testis
Kinin-Kallikrein pathway (WP5089)							
Degradation pathway of sphingolipids (WP4153)							
Glycosaminoglycan degradation (WP4815)							
nsp1 from SARS-CoV-2 inhibits translation initiation (WP5027)							
Lipid particles composition (WP3601)							
Non-homologous end joining (WP438)							
Cytoplasmic ribosomal proteins (WP477)							
Mitochondrial complex II assembly (WP4920)							
Proteoglycan biosynthesis (WP4784)							
Cholesterol biosynthesis with skeletal dysplasias (WP4804)							
Pentose phosphate metabolism (WP134)							
Mitochondrial complex I assembly model OXPHOS system (WP4324)							
Mevalonate pathway (WP3963)							
Oxidative phosphorylation (WP623)							
Electron transport chain: OXPHOS system in mitochondria (WP111)							

The 15 pathways have an effect size of > 0.3 indicating a change in protein abundance distribution between COVID-19 patients and controls. Pathways are shown in blue for a tissue if the protein abundance distribution shifted to the left, indicating a decreased activity of the pathway while pathways shown in red show increased activity. The stronger the color, the larger the effect.

### 3.5 Pathway activities per tissue

#### 3.5.1 Thyroid

The thyroid has the most changed pathways. There are 351 pathways that show significantly different activity. Among them, 50 pathways have the differences between COVID-19 and non-COVID-19 patients with an effect size > 0.3, see **Supplementary Table 1**. Most of them are more active in COVID-19 patients (36 out of 50 pathways). In the thyroid, the Kinin-Kallikrein pathway (WP5089) has the most reduction in pathway activity with the majority of proteins detected less abundant in COVID-19 patients. Among them, *F12* and *CPN1* are significantly different. Pathways depicting cholesterol, HDL and LDL metabolism (WP430, WP5109, WP4522, WP3601) are also among the top 10 pathways that have reduced activity in COVID-19 patients with significant reductions in APOA1, APOA2, APOC2, APOC3, APOB protein abundance. These proteins are apolipoprotein transporting and metabolizing phospholipid, cholesterol, and triglyceride. The post-squalene cholesterol biosynthesis and estrogen receptor (WP4804, WP2881) pathways have the most increase in activity with all proteins detected more abundant in COVID-19; the next most active pathways in the thyroid are omega-3/6 fatty acid synthesis pathways (WP4723) and mitochondria fatty acid synthesis pathway (WP4317) with all proteins detected more abundant in COVID-19. Sphingolipid metabolism pathways (WP1422, WP4726, WP4725) are also more active in COVID-19. The pathway depicting the altered glycosylation of *MUC1* as a promoter of chronic inflammatory conditions (WP4480) is more active with all proteins more abundant in COVID-19. Additionally, eight COVID-19 specific pathways from the WikiPathways and COVID-19 Disease Map show changed activity in the thyroid (see **Supplementary Table 1**).

#### 3.5.2 Lung

In the lung, 268 pathways have significant changes in COVID-19 patients. Among them, three pathways have an effect size > 0.3. The pathways describing the protein components in the 40S and 60S subunit of the translational factors (WP477 and WP5027) are active in COVID19 patients with the majority of proteins more abundant in COVID-19. Like in other tissues, the Kinin-Kallikrein pathway (WP5089) is also less active in the lung with five out of seven proteins, which are detected less abundant in COVID-19 patients.

#### 3.5.3 Heart

The heart has 50 significantly changed pathways. Only the Kinin-Kallikrein pathway (WP5089) has significant change with an effect size > 0.3. In the heart, this pathway is less active in COVID-19 patients with the majority of proteins detected less abundant in COVID-19.

#### 3.5.4 Kidney

In the kidney, there are 216 changed pathways with five pathways that have an effect size > 0.3. Most of them are less active in COVID-19. Pathways depicting lipid components (WP3601), the degradation of sphingolipids (WP4153) and the Kinin-Kallikrein pathway (WP5089) show reduced activity. The glycosaminoglycan degradation pathway (WP4815) depicting the lysosomal degradation of dermatan sulfate, heparan sulfate, keratan sulfate, and chondroitin sulfate is also less active in COVID-19. The only pathway that is more active in COVID-19 in the kidney is the pathway depicting the assembly of translational factors to initiate translation (WP5027) with all proteins in the pathway more abundant in COVID-19 patients.

#### 3.5.5 Liver

The liver has 215 significant changed pathways (p-value < 0.05). Among them, 10 pathways have an effect size > 0.3 between COVID-19 and non-COVID-19 patients. There are seven pathways that are more active in COVID-19 and three pathways that are less active in COVID-19 patients. Pathways depicting the assembly of translation factors from the 40S and 60S subunit (WP107, WP477, WP5027) have all proteins detected more abundant in COVID-19. Furthermore, DDX1 as a regulatory component of the Drosha microprocessor pathway (WP2942) depicting the posttranscriptional maturation of microRNA is more active with all proteins detected more abundant in COVID-19. Proteoglycan biosynthesis (WP4784) depicting the four main steps of protein synthesis in the endoplasmic reticulum and the Golgi apparatus to produce glycosaminoglycans is more active with the majority of proteins detected more abundant in COVID-19. Non-homologous end-joining (WP438) in which double-strand breaks in DNA are repaired throughout the cell cycle has all proteins detected more abundant in COVID-19. Mitochondrial complex II assembly (WP4920) depicting the assembly of complex II or succinate dehydrogenase (quinone) has the majority of proteins in the pathway more abundant in COVID-19 patients. The Kinin-Kallikrein pathway (WP5089) is less active in the liver with all proteins in the pathway less abundant in COVID-19 patients. The tyrosine metabolism (WP4506) pathway depicting the tyrosine degradation to the final product as fumarate and acetoacetate has all proteins detected less abundant in COVID-19 patients. Polyol pathway (WP690) depicts the two main enzymatic steps to metabolize unused glucose by first reducing glucose to sorbitol and then from sorbitol to D-fructose. D-fructose can then be metabolized in glycolysis. All four enzymes in this pathway were found with less abundance in COVID-19 patients than in non-COVID-19 patients.

#### 3.5.6 Spleen

The spleen has 311 changed pathways with 17 pathways having an effect size > 0.3, see **Supplementary Table 6**. The majority of pathways that have reduced activity the most in COVID-19 are the pentose phosphate pathway (WP134) and mevalonate pathway depicting cholesterol synthesis from acetyl-CoA (WP3963). Protein metabolism pathways such as purine metabolism (WP4792, WP4224) and cellular proteostasis (WP4918) are also among the most reduced pathways. The majority of pathways that have activity increase the most in COVID-19 are related to redox balance pathways such as electron transport chain: OXPHOS system in mitochondria (WP111), mitochondrial complex I/II/III assembly pathway (WP4324, WP4920 and WP4921) and oxidative phosphorylation (WP623).

#### 3.5.7 Testis

**Figure 6:**
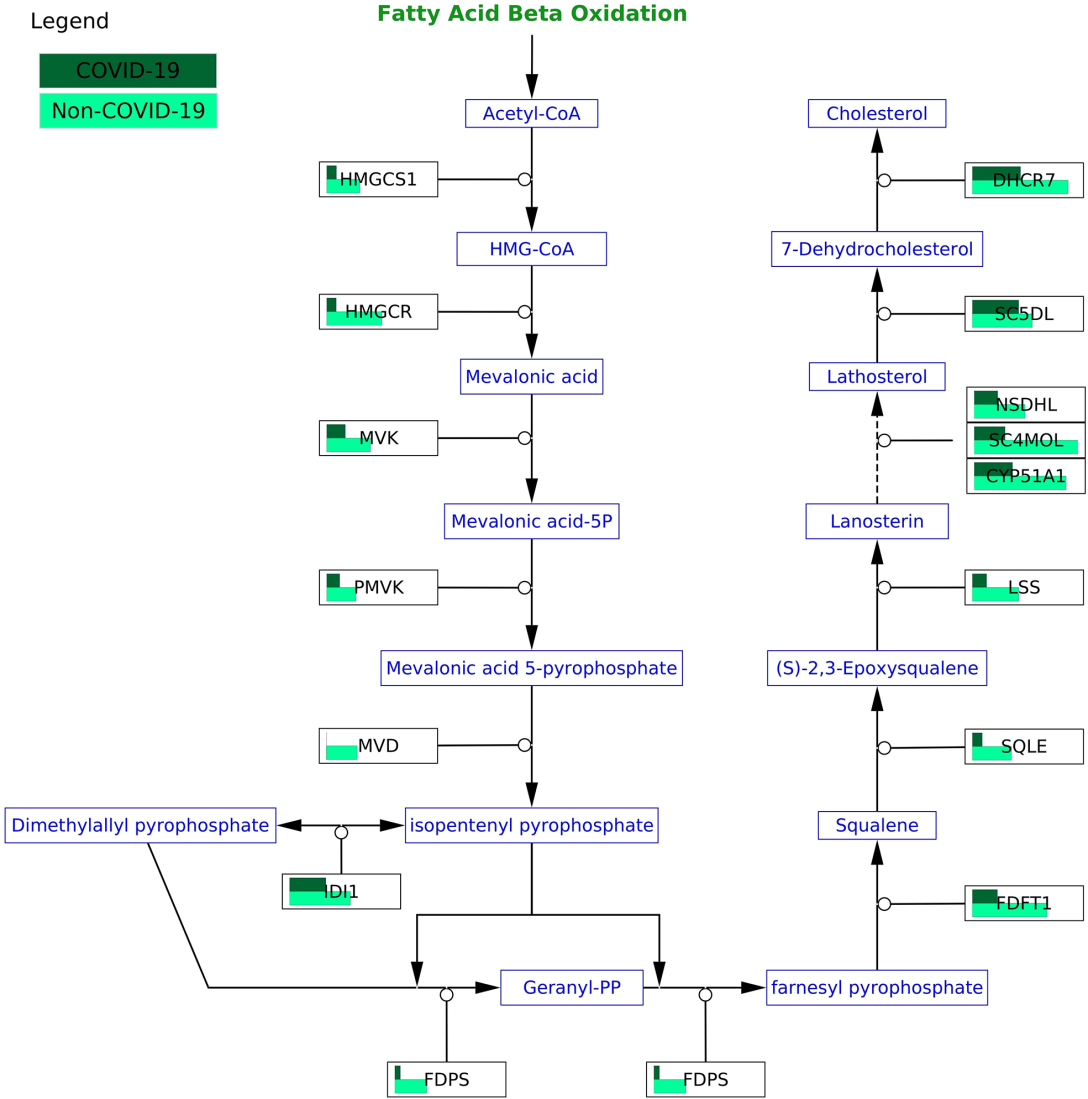
COVID-19 protein expression visualized in the cholesterol biosynthesis pathway (WP197) in the testis. All proteins in the pathway are less abundant in COVID-19 patients.

Of all 46 significant pathways in the testis, seven of them have moderate to high differences between COVID-19 and non-COVID-19 patients (size effect > 0.3). All these seven pathways were less active in COVID-19 patients. In the testis, complement cascade, cholesterol, and steroid-related pathways are less active in COVID-19. Namely, the cholesterol biosynthesis pathways (WP3963, WP197, WP4718, WP4804) in which cholesterol is synthesized from acetyl-CoA were less active with all proteins in these pathways lower in COVID-19 patients (**Figure 6**). The steroid biosynthesis pathway (WP496) in which steroids are generated from cholesterol and transformed into other steroids such as testosterone and estrone is less active with 4 out of 5 proteins detected lower in COVID-19 patients. Complement activation (WP545) pathway which depicts the classical pathway, the lectin pathway, and the alternative pathway generates the effector molecules of complement to recruit inflammatory and immunocompetent cells have all proteins detected (19 out of 22 proteins) reduced in COVID-19 patients.

## 4 Discussion

In this study, we investigated pathway activities in seven tissues: the thyroid, lung, heart, liver, spleen, kidney, and testis. Analyzing the publicly available proteomics dataset by Nie X et al. (2021) obtained from COVID-19 and non-COVID-19 autopsy samples (30), we identified 69 out of 640 pathways that have their activities significantly changed in COVID-19 patients. Our analysis showed a similar pattern in pathway activities in the testis and the heart with more pathways reducing in activities in COVID-19 patients. Other tissues: the spleen, kidney, lung, liver, and thyroid have similar pattern in pathway activities with each other with more pathways increasing in activities in COVID-19 patients.

In this study, we identify changes that are reported in COVID-19 patients such as the change in cholesterol, linolenic acid, and arachidonic acid metabolism, complement, and coagulation pathways in most tissues. We found a reduction in activity of cholesterol synthesis (WP3963) in the testis, thyroid, and spleen with a significant reduction in apolipoproteins (APOA1, APOA2, APOC2, APOC3, APOB) that are important for transporting cholesterol and lipids between cells to maintain normal lipid homeostasis (37). APOA1 or APOA2 deficiency can cause hypertriglyceridemia and in the long-term atherosclerosis (38–40). This reduction in these apolipoproteins and cholesterol might contribute to the damage leading to the failure in heart and kidney that has been observed in all the patients in the current cohort.

Of all changed pathways, the plasma kallikrein-kinin system (WP5089) has activity changes in the most tissues including the lung, thyroid, liver, heart, and kidney in COVID-19 patients. Kinin-kallikrein pathway is important during vascular inflammation citeLopatko2019. In this pathway, plasma kallikrein activates bradykinin, a pro-inflammatory peptide that then activates bradykinin 1 receptor (B1R) and bradykinin 2 receptor (B2R). B1R and B2R activation will increase the inflammation stage because it induces vascular permeability and neutrophil recruitment (41). Activation of B1R and B2R was speculated to be responsible for early pulmonary edema in COVID-19 patients (42, 43). Blocking B2R and inhibiting plasma kallikrein activity were proposed as a potential treatment for COVID-19 at the early stage (42, 43). In the initial phases of infection, Kinin-kallikrein pathway is highly activated being useful to recruit neutrophils (44). In our study, we found a reduction in Kinin-kallikrein pathway activity in most tissues (lung, spleen, testis, and thyroid) in COVID-19 patients. A study has shown an impaired function of Kinin-kallikrein activity with a reduction in bradykinin plasma level in severe COVID-19 patients (45). The authors hypothesized that the Kinin-kallikrein dysfunction related to disease severity in COVID-19 through inflammation, hypercoagulation, and lymphopenia.

Of all the tissue, we found the thyroid the organ with the most changed pathways in COVID-19. This is reasonable since the expression level of SARS-CoV-2 receptors (ACE2 and TMPRSS2) is higher in the thyroid than in the lung (46). With more receptors, more viruses can be expected in this tissue resulting in more alterations in pathways in the thyroid. In addition, the occupation of viruses on ACE2 can also impair other functions of ACE2. The loss of ACE2 can be harmful to the host sinceACE2 plays an important role as a counterbalance to the deleterious effects of Ang II (47). In the thyroid, we found an increase in activity of the angiogenesis *via* NRP1 (WP5065). The increase in angiogenesis may be induced by the dysfunction of ACE2 by SARS-CoV-2, which can contribute to the severity of COVID-19 (47, 48).

Reversible thyroid dysfunction was reported in COVID-19 (22). In this tissue, we found changes in lipid pathways, energy pathways, and many COVID-19 specific pathways such as RAS and bradykinin pathways in COVID-19, thrombosis, and anticoagulation. In the thyroid, the synthesis pathway of polyunsaturated fatty acids (PUFAs) such as omega-3, omega-6, and omega-9 was found to have increased activity. These PUFAs were shown to reduce inflammation and regulate immune cells in COVID-19 (49). We found a noticeable increase in activity of glycosaminoglycans degradation in the thyroid. However, this pathway showed decreased activity in the kidney. Impairment in glycosaminoglycans degradation resulting in the intralysosomal accumulation of undegraded products has been reported to cause cell, tissue, and organ dysfunction (50). Increases in circulating glycosaminoglycans and dysregulated glycosaminoglycans degradation were also observed in COVID-19 patients (51). We hypothesize that the reduced activity of glycosaminoglycans degradation can contribute to kidney failure in the current cohort.

The activity of translational factors pathways also increases with higher abundance in the components of 40S and 60S subunit in the lung, thyroid, and kidney in COVID-19 patients. SARS-CoV-2 is known to hijack the host ribosomal units for its own protein synthesis (52) With the increase in translation factors in the three most affected tissues in COVID-19: the lung, thyroid, and kidney, we hypothesize that the virus recruits them to increase their protein synthesis.

We did not find differences in immune pathways between COVID-19 and non-COVID-19 patients that are expected to be active in COVID-19 such as type 1 interferon signalling pathway (WP4868), TGF-beta signalling pathway (WP366), IL-1 or IL-6 signalling pathway (WP2332, WP364). These pathways were reported to contribute to the strong immune response leading to severe tissue damage in COVID-19 (53, 54). In the tissue proteomics dataset of the current study, we also found them to be among the active pathways in COVID-19 patients. However, these pathways are also found to be very active in non-COVID-19 patients in our analysis. This is a limitation in the dataset to have control samples from cancer patients instead of healthy people. Nevertheless, in our study we would like to focus on specific changes for COVID-19, using the current dataset and workflow we were able to identify such pathways. This will be helpful to interpret the nature of COVID-19.

The pathway activity statistics used overcomes some limitation of the commonly used over-representation analysis where only a small part of the data is used, e.g., only differentially expressed genes, or to eliminate false positive where the differentially expressed genes are not enough to influence the pathway, for instance in the case of IL-6 signaling pathway (WP364) and Type I interferon induction and signaling during SARS-CoV-2 infection (WP4868).

We indeed found changes in pathways that traditional statistical approaches such as over-representation analysis would exclude because these pathways do not contain differentially expressed genes. One example is the cholesterol biosynthesis pathway (WP197) in the testis with all 15 proteins in the pathway less abundant, but none are significant in COVID-19 patients. Studies have shown a reduction in cholesterol in severe COVID-19 patients (55). With all proteins less abundant in COVID-19 patients, it is reasonable to speculate that the activity of the cholesterol biosynthesis pathway is also reduced.

### 4.1 Limitations

The study of pathways is intuitive and provide detailed mechanistic insights into the disease. Nevertheless, the results can sometimes be misleading. Processes in human cells are interlinked and tightly regulated. The overlap and dependence between pathways are ignored and thereby it results in a more local and not system-wide analysis. In addition, the assumption of protein independence and ignoring the pathway topology when calculating the pathway activity means that every protein has an equal effect on the pathway activities independent of its position and role in the pathway.

Additionally, the pathway collections are still incomplete and for many of the measured proteins (60%) no related molecular pathway is available. Nevertheless, with the current data, we were able to identify pathways that are important in COVID-19 and pathways that have not been reported for COVID-19. WikiPathways and the COVID-19 Disease Map are growing, and new information is added continuously. The automation of the analysis will allow it to be easily rerun when novel pathway information has been added.

Another limitation of the current study comes from the incomplete nature of the proteomics dataset. While the dataset measures protein abundance for many proteins in seven different tissues, there are still many proteins in the pathway collection that are not measured in the dataset. It can either mean they were not present, or they were present but were not detected. Furthermore, the experimental setup is not ideal to study the processes affected in COVID-19 patients. The control samples are from cancer patients, which likely received treatment, and additionally, they might show strong alteration in cellular processes as well. Nevertheless, it is not possible to easily have biopsy samples for all the studied tissues such as lung or heart from healthy subjects. Importantly, compared to other omics data, proteomics is the closest data type to measuring protein expression levels making this dataset valuable to study pathway activities in the different tissues.

## 5 Conclusion

In this study, we analyzed a published proteomics dataset and identified changes in pathway activities in all seven organs tested. We highlight that SARS-CoV-2 does not only affect the lung but is a more systemic disease affecting many organs including the thyroid and testis which showed strong pathway activity changes in COVID-19 patients. The automated analysis increases reproducibility but also allows researchers to rerun the analysis when new pathway knowledge or experimental datasets becomes available.

## Data availability statement

Publicly available datasets were analyzed in this study. This data can be found here: doi: 10.1016/j.cell.2021.01.004iProX: IPX0002393000.

## Author contributions

NP and MK conceived the idea. NP conducted the data analysis. NP and MK interpreted the result. FT checked the code. NP drafted the manuscript. MK, FT and CE edited the manuscript. All authors contributed to the article and approved the submitted version.

## Funding

The project is funded by the ZonMw COVID-19 programme (Grant No. 10430012010015).

## Acknowledgments

We would like to thank Dr. Michiel Adriaens (Maastricht University) for his advice on the statistical approach.

## Conflict of Interest

The authors declare that the research was conducted in the absence of any commercial or financial relationships that could be construed as a potential conflict of interest.

## Publisher's note

All claims expressed in this article are solely those of the authors and do not necessarily represent those of their affiliated organizations, or those of the publisher, the editors and the reviewers. Any product that may be evaluated in this article, or claim that may be made by its manufacturer, is not guaranteed or endorsed by the publisher.
